# Evaluating hyaluronic acid dermal fillers: A critique of current characterization methods

**DOI:** 10.1111/dth.15453

**Published:** 2022-04-05

**Authors:** Ploymanee Wongprasert, Cécile A. Dreiss, Gillian Murray

**Affiliations:** ^1^ Institute of Pharmaceutical Science King's College London London UK

**Keywords:** cohesion, dermal fillers, hyaluronic acid, hyaluronidase, rheology, soft tissue augmentation

## Abstract

Soft‐tissue augmentation has gained much popularity in recent years. Hyaluronic acid (HA) based dermal fillers; a non‐permanent injectable device, can restore volume loss, fill fine lines and wrinkles and add curves and contours. HA based dermal fillers entered the non‐surgical treatment market in the late 1990s, however there is a lack of data and literature comparing the range of products and detailing the complexities of these products and how it relates to tissue performance. Measuring the physico‐chemical properties of these dermal fillers provide key parameters to predict their performance after injection into the body. This article reviews the currently reported methods and parameters used to characterize dermal fillers. The review of these methods and data from the literature provides a useful guide to clinicians and injectors in selecting the optimal product suitable for the needs of each patient.

## 1 INTRODUCTION

The human face ages as a product of complex microscopic and macroscopic volumetric changes. These changes are a product of the resorption of bony structures, gravity, subcutaneous fat redistribution, and skin damage.^1^ Dermal fillers are deployed to augment the face to meet the aesthetic concept of beauty, dictating that certain curves, contours, dimensions, and ratios are fulfilled in order to produce a conventionally attractive face, or to restore volumetric dimensions and hence youth in the aging face.^2^ Facial rejuvenation using soft tissue biodegradable fillers—a non‐permanent injectable device—provide an affordable and relatively safe procedure compared to permanent surgical cosmetic procedures.

The past decade has witnessed a dramatic rise in social media and online influencers. This, in turn, has contributed to the rise in popularity of aesthetic medicine. A recent survey suggests that the non‐surgical treatment market in the UK could be worth in excess of £3 billion within the next 5 years.^3^

Hyaluronic acid (HA) fillers are regarded as a class III medical device in the UK and not a medicine, due to the lack of an active pharmaceutical ingredient, where there is a pharmacological effect. Unlike medicines, medical devices such as dermal fillers have no legal requirement to provide safety and efficacy data, or how they perform in comparison to other market brands.^4^ In Europe manufacturers are expected to complete a certificate of conformity, in line with the Medicine Device Regulations general safety and performance requirements. This is mainly linked to manufacturing and risk assessment. As the UK is no longer part of Europe, the certification of devices is changing, and CE will become UKCA.^5^ The FDA require more detailed information linking to safety and effectiveness of the product, therefore there is more rigor applied to approving these products for use.^6^ As HA fillers are not medicines, claims can be made with little to no evidence to substantiate them. The lack of regulation in the HA filler market, and the financial value of the industry, means there is a lack of objective guidance to clinicians when choosing HA fillers.

Understanding the physico‐chemical properties of dermal fillers is important to make informed product selection. HA fillers differ from each other due to the different crosslinking technologies used, which aim at tuning the mechanical properties to the target tissue and the biological outcome after injection. During the injection process, gels are subjected to shear stress and vertical compression/elongation forces, which cause the filler to deform. Dermal fillers under low stress are gel‐like materials, but flow under increasing shear stress, to different extents depending on their specific manufacturing conditions and composition.^7^, ^8^

Key questions are: can these products correctly mimic the soft tissue or bones they are replacing? Which measurable physico‐chemical parameters can be used to predict the long‐term performance of fillers? How do stress, deformation, temperature and enzymatic degradation affect their properties over time?

While addressing these questions should be a long‐term endeavor, the objective of this review is to present the state of research on cross‐linked HA dermal fillers, focusing on current methods and parameters reported in the literature, to evaluate whether these measurements can be used as a valid measure of how HA fillers may perform in vivo.

## 2 REVIEW METHODOLOGY

A literature search was conducted using Embase and Google Scholar to identify suitable research papers. Articles from the last 20 years were included. References from each article were reviewed to identify any papers of further interest. The aim was to identify papers reporting the physico‐chemical and rheological properties of HA dermal fillers, specifically FDA‐approved HA fillers and Revolax (a filler from the Korean market that is widely used globally as a less costly product). No information regarding Revolax was found in scientific publications, therefore the manufacturers device patent was accessed to provide product information. It was necessary to search for patent information for Teoxane RHA, Restylane OBT, and Juvaderm Vycross technologies to establish the degree of cross linking. This measurement often differs in research literature therefore manufacturers patent application was used to find this value.

Papers were analyzed and critiqued, to review the methodologies used to investigate these HA properties in seminal literature. The search used the following key words: hyaluronic acid, dermal fillers, soft tissue augmentation, rheology, hyaluronidase, cross‐linked hyaluronic acid.

## 3 PROPERTIES OF HA RELEVANT TO APPLICATION IN DERMAL FILLERS

### 3.1 HA's chemical structure

**FIGURE 1:**
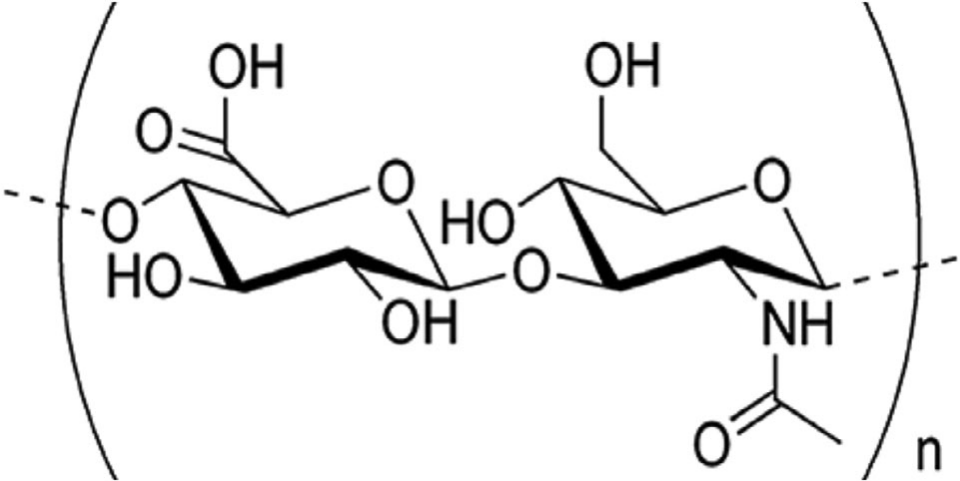
Hyaluronic acid monomeric unit

HA is an abundant polysaccharide of the extracellular matrix,^9^ consisting of repeating units of the sodium salt of d‐glucuronic acid and d‐N‐acetylglucosamine, linked together linearly by a β‐1,4 glycosidic bond,^10^ resulting in the disaccharide structure shown in Figure 1.

### 3.2 Crosslinking and degree of crosslinking

Upon dissolution in water, HA forms highly viscous solutions. These solutions are not suitable for use in dermal fillers as they will not stay in place at the injection site and also be rapidly degraded by the enzyme hyaluronidase,^11^ resulting in a limited residence time.

**FIGURE 2:**
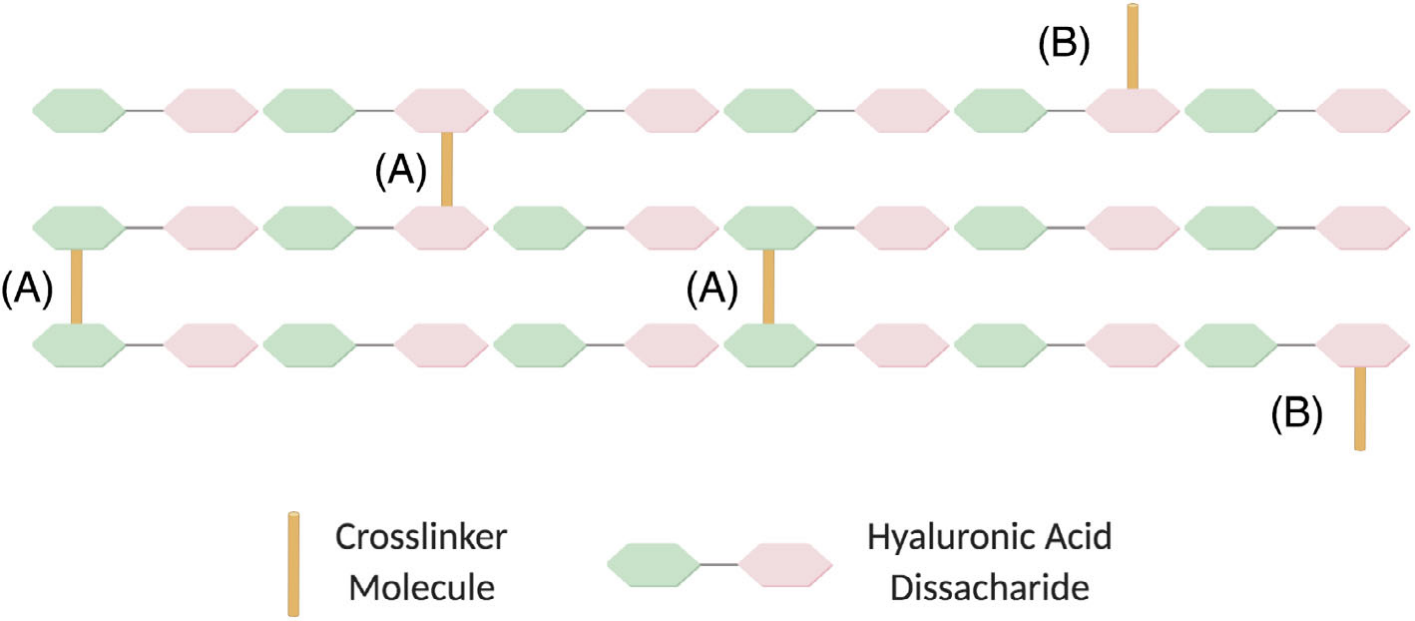
Illustration of HA chains bound by (A) a crosslinker molecule linking two chains (creating an elastic junction) and (B) a crosslinker linked to only one chain (pendant end). HA, hyaluronic acid

In order to achieve the required stiffness and persistence, a number of modifications and processing steps must be carried out. Specifically, crosslinkers are used to connect HA polymer chains together to create a network (Figure 2), and transforming the viscous liquid into a gel.^12^ The crosslinked dermal fillers available in the UK today are mostly crosslinked with 1,4‐butanediol diglycidal ether (BDDE).

The crosslinking technology differs across brands and has an impact on the properties of the resulting gel. During the crosslinking process, the crosslinker becomes permanently bound to the HA chains via strong covalent bonds. The crosslinker can either bind at both ends, creating a diol that is stable and does not react further, or bind only at one end, resulting in a “pendant” end^13^, ^14^ (Figure 2), the ratio of which will affect the stiffness of the final gel.

The degree of crosslinking (CrD)^14^ can be quantified by the ratio of the crosslinker molecules that form crosslinks to the number of HA disaccharides. In Figure 2, for example, the CrD is 3/36 = 0.083 (8.3%). If all other factors are equal, the higher the CrD, the longer the residence time of the gel under the skin. High degrees of crosslinking could lead to very high stiffness and reduced hydrophilicity of the HA polymer chains, in turn compromising the lifting capacity of the gel. Additionally, exceeding this threshold could affect the biocompatibility of the product, which could lead to an immune response or an adverse reaction to the gel. There is, however, no established higher threshold value for CrD.

In the UK, medical devices such as dermal fillers are only required to have a CE marking,^15^ an indicator of the product's compliance with EU legislation. Quality control of the product is not a requirement, this means that the presence of unreacted or residual crosslinker molecules can remain in the final product, which can be toxic in high concentrations,^16^ potentially leading to harmful adverse reactions.^17^

### 3.3 Crosslinking technology

The proprietary crosslinking technology of the HA gels influence the gel properties in many ways. Dermal fillers discussed in this review have been manufactured using three different methods: Resilient Hyaluronic Acid (RHA®),^18^ Vycross®,^19^ and Optimal Balance Technology (OBT®)^20^ (Table 1).

**TABLE 1:** Main dermal filler products discussed in this review, all commonly used in the UK

Brand	Product	Concentration of HA (mg/ml)	Crosslinking technology	Percent crosslinking	Degree of correction and (site of application)^a^
Revolax	Fine	24^21^	Ursolic acid encapsulation^21^	Unreported	Fine, superficial and lip submucosa (crow's feet, neck, and wrinkles)
	Deep	24^21^	Ursolic acid encapsulation	Unreported	Fine to medium, superficial to mid dermis (forehead lines, tear troughs)
	Sub‐Q	24^21^	Ursolic acid encapsulation	Unreported	Moderate to severe, mid to deep dermis (facial contours, cheek, nose, chin)
Teoxane	Redensity II RHA 1 RHA2	15^43^ 15^44^ 23^44^	Standard RHA^33^ RHA	Unreported 1.9%^45^ 3.6%^45^	Fine, superficial (tear trough, periorbital regions) Dynamic perioral rhytids and barcode lines Dynamic lip volumization
	RHA3	23^44^	RHA	6.02%^45^	Moderate to severe, mid to deep dermis (deep wrinkles)
	RHA4	23^44^	RHA	6.85%^45^	Moderate to severe, deep dermis to subcutaneous (cheek, chin, temples)
	Ultra‐deep	25^14^	Standard	10%^45^	Severe, deep dermis (chin, cheekbones, jawline)
Juvéderm	Vobella	15^44^	Vycross^44^	6.61%^45^	Fine to medium, superficial to mid dermis and lip submucosa (lateral canthal lines, tear troughs, lips)
	Volift	17.5^44^	Vycross	7.73%^45^	Medium, midface volume loss and cheek augmentation, deep dermis to subcutaneous (cheek, facial contours, lips)
	Voluma	20^44^	Vycross	7.36%^45^	Medium, midface volume loss and cheek augmentation, deep dermis to subcutaneous (temple and lateral brow, medial brow, cheek, jawline)
	Volux	25^44^	Vycross	9.4%^45^	Moderate to severe, deep dermis (temple and lateral brow, medial brow, cheekbones, jawline)
Restylane	Fynesse	20^44^	OBT^32^	0.1%–5%^19^	Fine to medium, superficial to mid dermis and lip submucosa (nasolabial folds, lips)
	Volyme	20^44^	OBT	0.1%–5%^19^	Moderate to severe, deep dermis to subcutaneous (temple and lateral brow, cheek, chin)
	Refyne	20^44^	OBT	6%^30^	Moderate to severe, mid to deep dermis (lateral canthal lines, tear trough)
	Defyne	20^44^	OBT	8%^30^	Moderate to severe, mid to deep dermis (temple and lateral brow, cheek, medial brow, jawline)

Abbreviation: HA, hyaluronic acid.

^a^ Based on product information provided by manufacturers.

Revolax's crosslinking technology involves the encapsulation of ursolic acid,^21^ a natural wax found in fruit peels, within the HA polymer network. This technology claims to maintain a long‐term durability without increasing the crosslinking density. RHA® is the technology used by Teoxane, whereby the gels are stabilized by natural and chemical crosslinks to produce gels with long chains of HA. Their range uses 1.9%–4.0% BDDE,^18^ which is relatively low compared to other brands, and they also have differing concentrations of HA. Vycross®, used in the latest Juvéderm range, is formulated with a mixture of high molecular weight HA and low molecular weight HA, with a higher ratio of the latter, linked with BDDE at both ends.^19^ Finally, OBT® is the technology used by Restylane. Products in this range have the same HA concentration but achieve a range of gel firmness by varying the degree of crosslinking.^20^

### 3.4 Manufacturing process: fragmentation

The manufacturing process used to produce HA dermal fillers involves breaking down the initial crosslinked gel into smaller HA gel fragments or particles.^16^ This process allows the gel to flow through a needle for injection under the skin. After fragmentation, the gels may still be too stiff and hence resistant to deformation and potentially difficult to inject. In order to overcome this, some manufacturers introduce un‐crosslinked HA as a lubricant to reduce the strength of the gel during injection^16^ (and therefore the force required for injection). While this un‐crosslinked HA aids the smooth injection of the gel, it has a short residence time under the skin and thus does not contribute to the persistence of the gel at the site of injection.

## 4 METHODS CURRENTLY USED TO CHARACTERIZE HA DERMAL FILLERS

### 4.1 Dynamic rheology – oscillatory frequency sweeps

HA dermal fillers are viscoelastic materials, which means that they display both a viscous (irreversible deformation) and elastic (reversible deformation) response when a force is applied. Rheological parameters provide a measure of how the ability of a dermal filler to resist deformation, which is relevant to key parameters such as injectability, lifting capacity (profile of the filler after injection) and residence time. Oscillatory (or dynamic) frequency‐sweep tests are employed to determine the material's overall resistance to deformation, G*; the elastic modulus, *G*′; the viscous modulus, *G*″; and the phase angle, *δ*.^22^

In practice, a small amount of the dermal filler is placed between two plates, and one of them rotated by a small angle, creating a shear stress (force per unit area) and inducing deformation (strain). G* is defined as the ratio of the shear stress to the shear strain (the ratio of the displacement to the height of the sample, or gap). For instance, a higher extent of crosslinking will require a greater stress to achieve the same displacement (or will be deformed less for the same stress applied), resulting in a higher modulus value, characteristic of a “stiffer” gel.

Numerous studies report dynamic frequency‐sweep tests conducted in oscillatory mode, using parallel‐plates^13^, ^14^, ^23^, ^24^ or cone‐and‐plate geometries.^13^, ^25^, ^26^ In this type of experiment, the frequency is typically varied over a few decades of frequency (typically 0.01–100 rad/s, often a narrower range). Most studies have reported measurements at temperatures of 25°C,^13^, ^23^, ^25^, ^26^ however, physiological temperature is more relevant to the clinical application and this may need to be taken into consideration for future work.^24^, ^27^

**FIGURE 3:**
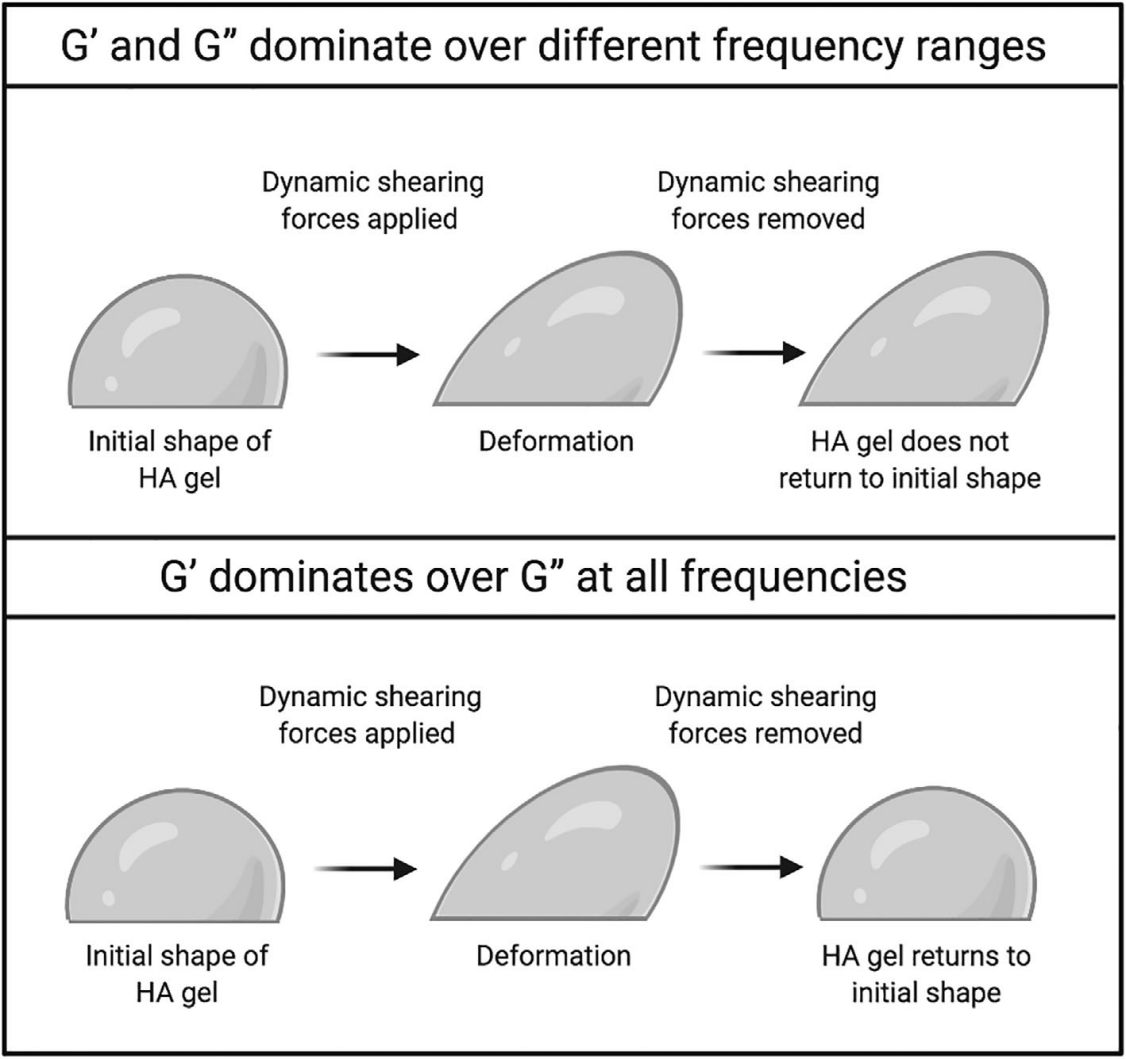
Schematic representation of how the elastic (*G*′) and viscous (*G*″) moduli reflect the capacity of the gel to deform and flow and impact its shape when exposed to shearing forces (adapted from^25^)

The ratio of *G*″ and *G*′, or tan *δ*, reflects the relative magnitude of the viscous and elastic modulus. A predominantly elastic gel (low tan *δ*) deforms under the action of stress and recovers its shape after removal of the force (Figure 3), while a gel where viscosity dominates (usually at low frequencies, i.e., long times) deforms but also flow (Figure 3). As a result, the phase angle *δ* is often linked to the capacity or otherwise of a product to migrate, and Revolax claimed that the low phase angle of their product relates to limited product migration from the site of injection.^28^

Only in an “ideal” gel (permanent cross‐links, monolithic gel) are *G*′ and *G*″ independent of frequency, with *G*′ dominating over all frequencies. For typical HA fillers, due to the presence of un‐crosslinked chains and the particulate nature of the gels, this is not the case: both *G*′ and *G*″ are dependent on the frequency and therefore only a full frequency sweep curve enables meaningful comparisons between different products. In the current literature, many studies only report the value of *G*′ at one frequency, which gives an incomplete characterization of the sample.

Across the studies reported in the literature, it is clear that the viscoelastic properties vary substantially between products. Many factors can explain these differences: HA concentration, molecular weight, crosslinking technology and amount of free HA present in the final product.^23^, ^29^ Many authors report crosslinking density as the factor with the greatest influence on the rheological properties, specifically the stiffness of the gel.^25^, ^30^, ^31^

A comparative study of 11 standard products sold in the UK (measured in our laboratory) showed for all a predominantly elastic behavior (*G*′ was above *G*″ across all frequencies), except for the products intended for more superficial tissues, such as under‐eye and tear trough treatment (Teoxane's Redensity II) and superficial lines (Revolax' Fine), in line with their intended target application. Table 2 shows a partial summary of this data, with *G*′ values extracted at 10 rad/s. For some of the brands (Revolax and Teoxane), a wide range of values are obtained across the range of fillers, which provides many clinical indications for different depths of site of injection. On the other hand, Juvéderm's Vycross range exhibits a narrower range of *G*′ values, on the “stiffer” side. However, only the full frequency sweep curves provide a true “fingerprint” of the rheological behavior, with values of *G*′ and *G*″ displaying frequency‐dependence (as expected for a particulate material that is not a fully cross‐linked gel). The full range of frequencies is however rarely reported in dermal filler literature, with generally one value of *G*′ quoted at an arbitrarily chosen value of frequency.

**TABLE 2:** *G*′ values of 11 dermal fillers measured at 10 rad/s

Product	*G*′ (Pa)
Teoxane Redensity II	80
Teoxane RHA3	261
Teoxane RHA4	346
Teoxane Ultra‐deep	366
Juvéderm Vobella	239
Juvéderm Volift	350
Juvéderm Voluma	493
Juvéderm Volux	677
Revolax Fine	167
Revolax Sub‐Q	246
Revolax Deep	344

After injection, HA fillers are subjected not only to dynamic shear stresses due to facial movements, but also compressional stresses. However, rheological measurements (which apply shear stress) dominate the dermal fillers literature, apart from a few exceptions.^25^ To understand how a product will persist in the tissue when under muscular forces (such as when speaking, smiling or eating), both the dynamic shear stress and compression stress of the gel should be measured.^7^ Both *G*′ and *E*′ (the Young or compression modulus) provide complementary information and must be within a suitable range to withstand these facial forces.^25^

### 4.2 Swelling

When in solution, HA chains expand due to their affinity with water,^16^, ^22^; when the polymer chains are crosslinked, this results in swelling of the gels. Swelling is an essential parameter for dermal fillers as it is directly related to how the filler will expand at the site of injection. A gel's capacity to swell is dependent on factors such as the concentration of polymer, the degree of crosslinking and the process by which the gel was hydrated. A strong correlation between swelling factor and cohesion has also been reported: the further away the product is from equilibrium swelling (the point beyond which the product phase separates between a polymer‐rich and a water‐rich phase), the more cohesive the product is.^32^

Limited swelling is expected in tighter gel networks (higher extent of crosslinking, hence higher *G*′),^13^, ^23^, ^32^ leading to a lower propensity for fluid uptake. However, while this relationship may be true within a given filler series, it may break‐down across series due to different crosslinking technologies.^33^

In a typical swelling test, an aqueous solution is added to a precisely weighted quantity of dermal filler and left for a given period of time. The resulting mixture is then centrifuged, the supernatant removed, and the resulting hydrated gel weighted. The swelling ratio can be calculated using the following equation:

$$\mathrm{SR}\%=\left(\frac{W_{s}-W_{d}\;}{W_{d}}\right)\times 100$$

where, *W*_*s*_ is the mass of the swollen gel and *W*_*d*_ is the mass of the dry gel.

Kablik et al.^13^ have proposed a method whereby samples were diluted to different extents, and their dynamic rheological properties measured. The phase angle, *δ*, plotted against the percentage dilution gives a “dilution durability,” which can be understood as the equilibrium point before phase separation occurs. Size Exclusion Chromatography along with Multi‐Angle Laser Light Scattering have also been used to determine the gel‐to‐fluid ratio (a ratio of insoluble HA to soluble HA). A strong correlation between “dilution durability” and gel‐to‐fluid ratio was observed: the more fluid (i.e., soluble HA) in the product, the higher extent to which the product can swell before phase separation occurs.

The swelling factor (SwF) is defined by *V*/*V*_0_ where *V*_0_ is the initial volume of the gel and *V* is the fully swollen volume.^14^ Table 3 shows data obtained on the Restylane's OBT filler range. The reveal some correlation between SwF and *G*′: the lower the *G*′, the higher the SwF. However, it was not possible to establish whether the degree of crosslinking (CrD) correlates with swelling; as explained above, this correlation often breaks down across different brands due to different technologies used.

**TABLE 3:** Degree of crosslinking (CrD), swelling factor (SwF) and elastic modulus (*G*′) for Restylane's OBT® range of fillers

Product	CrD (%)	SwF (ml/g)	*G*′ (Pa)
Fynesse	0.1–5	17.2	10
Volyme	0.1–5	7.3	150
Refyne	6	9.7	47
Defyne	8	6.4	260

*Note*: HA concentration is 20 mg/ml across the range. *G*′ values were extracted at a frequency of 0.1 Hz. Data reproduced from Reference 32.

Abbreviation: HA, hyaluronic acid.

### 4.3 Enzymatic degradation

HA is a substrate for the enzyme hyaluronidase: the enzyme cleaves HA molecular strands into smaller oligosaccharides, making them susceptible to metabolism and clearance from the body.^30^ Increasing the degree of crosslinking is expected to reduce the propensity to enzymatic degradation and achieve longer residence.^34^

In addition to the degree of crosslinking, HA concentration also influences the rate of degradation. Many authors have reported this trend through qualitative in vitro studies,^35^ quantitative in vitro studies^36^, ^37^ animal models,^38^ and clinical testing of human subjects.^39^

To measure enzymatic degradation, typically, a solution of hyaluronidase is mixed with the gel. The mixture is centrifuged, the fluid phase filtered out and the remaining gel weighted. This process can be performed over several time points, until the gel is completely degraded.^30^ This measure is important to understand how a given filler will respond to the injection of hyaluronidase, routinely used in case of occlusions.

### 4.4 Cohesion tests

Cohesion is a parameter often mentioned in the literature of dermal fillers, which, while intuitively quite accessible, remains poorly characterized. According to Micheels et al.,^33^ gel cohesion is the sum of internal forces that unite the solid and liquid phases; if a gel is cohesive, then the gel remains monophasic when placed in an aqueous solution. Fagien et al.^23^ relate gel cohesion to HA concentration and the crosslinking technology used. Edsman et al.^32^ recognize that the property is difficult to measure and subjective. They also argue that measurement of this property before injection into the tissue is not relevant as the gels contain free HA that will dissolve once injected and do not remain at the injection site, making the gels more cohesive after injection.

A simple test was carried out by Micheels et al.^33^ to determine gel cohesion, whereby a small amount of the HA gel is mixed with a saline solution and observed under a microscope. If long strands are observed under magnification, then the gel is deemed cohesive and if it breaks down into particles then it is non‐cohesive. Their study across different crosslinking technologies found that RHA, Vycross, and OBT gels all resulted in the breakdown of the gel network, as large or fine grains were observed. What this suggests is that these gels do not remain monophasic and will not remain evenly distributed after injection, ending up instead into clumps or pools of material. This, however, may not necessarily be an issue, depending on the desired outcome. Falcone et al.,^26^ for instance, consider cohesiveness to be undesirable, as highly cohesive HA gels are generally non‐crosslinked dilute solutions which have a shorter residence time in the tissue and low elasticity.

Both Edsman et al.^14^ and Fagien et al.^23^ carried out a drop weight test to measure cohesion. In this test, HA gels are extruded at a constant speed through a defined vertical orifice. The droplets are collected, and an average drop weight is measured.^23^, ^32^ Products with higher average drop weights correlated with lower *G*′ values and were deemed more cohesive. It is important to note that these trends were only seen within the same crosslinking technologies. Qualitative tests were also performed by Edsman et al.,^14^ where sensory analysis was used to determine the perceived cohesion, showing a good correlation with the drop weight test.

A qualitative assessment of cohesivity has also been reported by La Gatta et al.,^29^ using a protocol originally proposed by Sundaram et al.,^40^ where the gel is stained by toluidine blue. A small amount (ca. 1 g) is then extruded from a syringe into a 1 L beaker of water, with continuous stirring. The droplets are filmed, and images evaluated at specific times, and a value of cohesivity assigned.

**FIGURE 4:**
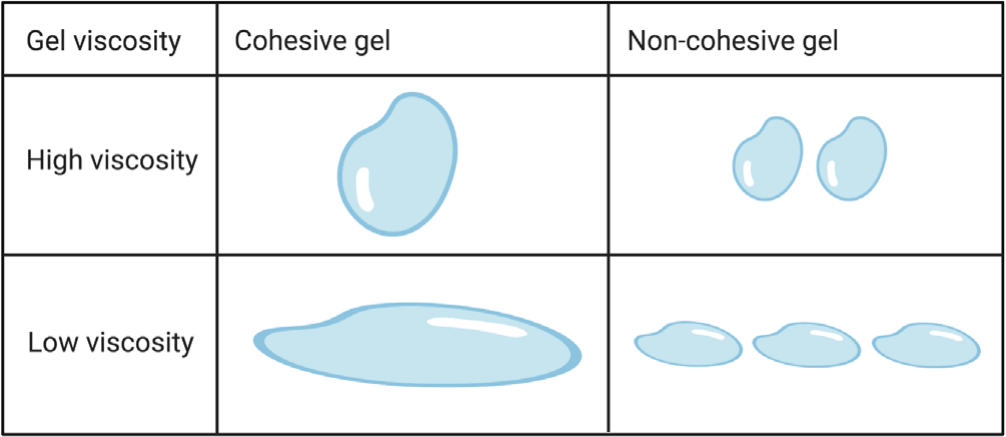
Schematic representation of the relationship between cohesivity and viscosity of a HA gel to show its capacity to remain or spread at the injection site (adapted from^25^). HA, hyaluronic acid

Both the viscosity of the gel and its cohesivity (Figure 4) are likely to determine how the gel distributes within the tissue: in order to obtain good tissue integration, both of these parameters need to be carefully considered. If the tissue is firm (such as cheek bones or the jaw line),^41^ a high viscosity gel that is highly cohesive can be molded by massaging the area after injection, allowing more precise placement without fragmentation. Instead, a HA gel with low viscosity may be more suitable for superficial indications (such as fine lines and wrinkles) as the product will flow and spread more, giving a profile more akin to the area that is being treated.^25^

### 4.5 Particle size and particles per ml

HA dermal fillers are not “bulk” gels but are constituted by crosslinked HA particles, resulting from fragmentation.^26^ The process of sizing down the gel mass is performed by passing the gel through a series of sieves and screens.^16^ Depending on the sieving method, the various products will have a distinct average gel particle size and shape, which will impact the final product's performance.

For dermal fillers, there is a maximum particle size beyond which the gel cannot be extruded easily and may clog the needle during injection.^13^ On the other hand, larger HA particles have a limited total surface area for enzymatic breakdown, while it is smaller for HA particles, which therefore degrade faster.

A good trade‐off can be achieved by breaking down the mass by a homogenization process. This process results in a broader distribution of gel particle sizes than obtained by sieving, and “softer” gels with lower *G*′ values.^36^

Particle size may be linked to the residency of the HA filler in situ: it is generally thought that the larger the particle size, the longer they reside in the tissue.^42^ However, the link between particle size and important parameters such as the elastic and viscous moduli, or long‐term performance, is not obvious.^26^

### 4.6 Extrusion force

Extrusion force is another important parameter of high clinical relevance as it relates to the force that the physician must apply to allow the HA filler to flow through the needle.^41^

**FIGURE 5:**
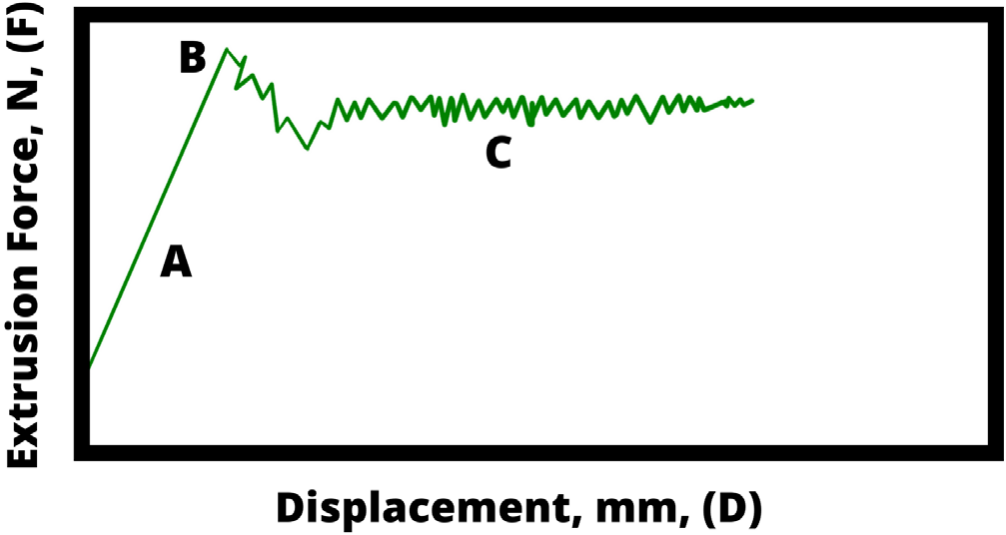
Extrusion curve of HA dermal filler with yield point, where extrusion force, *F* is plotted as a function of displacement, *D*. HA, hyaluronic acid

Figure 5 represents a typical extrusion curve, where the force required to push the gel from the syringe, *F*, is plotted as a function of the displacement of the syringe, *D*. If the syringe is pushed at a constant rate, a linear relationship is observed between force and displacement (slope A); this is known as the linear elastic regime. This slope is proportional to *G*′, therefore gels with higher *G*′ values will require higher pressure on the syringe.

The peak labeled B in Figure 5 is the yield point; this is where the curve becomes nonlinear and the sample starts to (plastically) deform; beyond this point, the gel begins to flow from the needle into the injection site, and the force drops. Finally, the region labeled C is where the viscous regime dominates, and force is nearly constant with displacement: the injection of the filler is smooth and at a steady rate. As the viscous regime dominates here, the force needed to extrude the gel heavily relies on the viscosity of the filler.

This behavior has a huge impact on clinical outcome: if the clinician stops applying a steady force at any point, the whole process has to be repeated to reach the viscous regime again. Starting and stopping may result in an uneven distribution of product, creating an uneven and undesired outcome.

There are no quantitative data reported on the extrusion force profile. While this aspect is linked to practice, it would be useful to produce quantitative data to better understand the optimum force profile required to extrude a given filler from the syringe in an even and smooth manner.

## 5 LINKING EXPERIMENTAL PARAMETERS TO PERFORMANCE

The clinical performance of a HA based crosslinked dermal filler is dictated by their physico‐chemical properties. Depending on the specific clinical indication, many parameters need to be considered to optimize the formulation or to select the most appropriate product.

Within the range of parameters reviewed in this article, the elastic modulus, *G*′, is the most widely reported and perhaps most relevant parameter; strong correlations have been established between *G*′ and other parameters such as swelling, the degree of crosslinking and cohesion.^23^, ^25^, ^26^

“Stiffer” gels possess higher *G*′ values; these gels swell less, tend to be classified as more “cohesive,” and, as a result, are also more resistant to enzymatic degradation. These types of gels, such as Revolax Sub‐Q (*G*′ = 281 Pa) (unpublished data), Teoxane RHA4 (*G*′ = 296 Pa),^23^ Juvéderm Volux (*G*′ = 307 Pa),^23^ and Restylane Defyne (*G*′ = 260 Pa)^23^ (all values taken at 0.1 Hz), are more suitable for areas where bony structures need to be replicated; these stiffer gels are able to resist the high shear forces found under the muscles).^41^ However, high cohesion is not always a desirable property, as facial movements could result in a “bunching up” effect where the gels aggregate into clumps. The high cohesion could prevent the gel from spreading, thus producing an uneven contour.

On the other hand, “softer” gels, with lower *G*′ values, such as Revolax Fine (*G*′ = 93 Pa) (unpublished data), Teoxane Redensity II (*G*′ = 37 Pa) (unpublished data), Juvéderm volbella (*G*′ = 159 Pa),^23^ and Restylane Fynesse (*G*′ = 10 Pa)^23^ (all at 0.1 Hz). These gels tend to be formulated with lower HA concentrations and/or lower degrees of crosslinking.^30^ Due to the lower extent of crosslinking, possibly higher amount of free HA, resulting in a looser gel network, they are more susceptible to enzymatic degradation, and will be more readily eliminated by the body. These fillers are more suited to finer corrections and can provide a more natural feel when injected, such as for less dynamic wrinkles (tear troughs, soft tissue found in lips and the periorbital region). However, these gels tend to have a shorter residence time in the tissues.^34^

The drop weight test reported in various studies considers that the lighter the average drop weight, the less cohesive the gel is.^23^, ^32^ This characteristic also correlates with gels with lower *G*′ values. Less cohesive gels have looser polymer networks, thus once extruded from the needle will drop quicker, therefore producing smaller and lighter drops. The less cohesive nature of these gels is more likely to produce smoother contours once injected. However, this could also pose a problem of gel migration from the injection site.

A property that also correlates with a low *G*′ value is the swelling factor: increased gel fluid uptake is usually observed for gels with lower *G*′ values (unpublished data). This could pose an issue for clinical applications because if gels with low *G*′ are recommended for superficial applications, such as in the tear trough, the swelling of the gel could cause a convexity, which is an extremely undesirable outcome. Clinicians must take into account a gel's propensity to swell and warn patients that swelling will subside or inject a smaller amount of filler, because swelling will contribute to the volume injected.

Beyond dynamic rheological measurements, the lack of standardized measurements is a challenge when attempting to compare physico‐chemical properties from different fillers. Differences in crosslinking technologies, HA concentration, particle size and degree of crosslinking across brands make the establishment of universal correlations challenging. More importantly, there is yet no reported physico‐chemical parameters that take into consideration clinical and anatomical effects on the overall long‐term performance of the gel once injected into the body.

When choosing a suitable product, it is important to consider the site of injection, including which anatomical facial layer. Different areas and layers of the face are subject to different magnitudes, frequencies and type of forces, which need to be considered to achieve the optimal outcome. There are two types of forces that govern the mechanical stresses within the face: intrinsic forces (dynamic tensions that occur between tissues within the face, such as bone, muscles, fat and skin) and extrinsic forces (environmental forces that result from daily activities: sleep, nutrition, exercise, etc.).^41^ Steady‐state shear rheology (measuring viscosity as a function of shear force) can provide a measure of how gels will react to these forces, however more work needs to go into modeling the shear, compression and torsion forces each facial zone may experience and measure the properties of fillers under conditions that better mimic them.

As mentioned above, the phase angle obtained from dynamic rheology is another parameter often quoted to advertise fillers which claim to have a “low percentage to migrate,” however no conclusive evidence has been found across the literature that supports this claim. Better methods must be put forward to measure the migration of a product.

## 6 CONCLUSION

A growing body of literature reports experimental data on HA dermal fillers, however, correlation within clinical application and long‐term performance is lagging behind.

Overall, the methods described in this review and commonly reported on fillers do not predict the long‐term performance of the gels. However, a large set of physico‐chemical parameters can act as an indicator of performance, which, alongside clinical experience, is extremely helpful in guiding clinicians in choosing the optimal product for a specific application.

Future work should focus on establishing standardized experimental protocols and link them to clinical data.

## CONFLICT OF INTEREST

We declare no conflict of interest and no funding sources.

## AUTHORS CONTRIBUTIONS

**Ploymanee Wongprasert**: Conceptualisation, investigation, visualization, writing, original draft. **Cécile A. Dreiss**: Supervision, conceptualisation, investigation, writing, review and editing. **Gillian Murray**: Supervision, conceptualisation, investigation, writing, review and editing.

## Data Availability

Data sharing is not applicable to this article as no new data were created or analyzed in this study.
